# Human Dermal Fibroblasts Demonstrate Positive Immunostaining for Neuron- and Glia- Specific Proteins

**DOI:** 10.1371/journal.pone.0145235

**Published:** 2015-12-17

**Authors:** C. J. Janmaat, K. E de Rooij, H Locher, S. C. de Groot, J. C. M. J. de Groot, J. H. M. Frijns, M. A. Huisman

**Affiliations:** 1 Department of Otorhinolaryngology and Head & Neck Surgery, Leiden University Medical Center, Leiden, the Netherlands; 2 Percuros B.V., Enschede, the Netherlands; 3 Department of Radiology, Leiden University Medical Center, Leiden, the Netherlands; Hungarian Academy of Sciences, HUNGARY

## Abstract

In stem cell cultures from adult human tissue, undesirable contamination with fibroblasts is frequently present. The presence of fibroblasts obscures the actual number of stem cells and may result in extracellular matrix production after transplantation. Identification of fibroblasts is difficult because of the lack of specific fibroblast markers. In our laboratory, we isolate and expand neural-crest-derived stem cells from human hair follicle bulges and investigate their potential to differentiate into neural cells. To establish cellular identities, we perform immunohistochemistry with antibodies specific for glial and neuronal markers, and use fibroblasts as negative control. We frequently observe that human adult dermal fibroblasts also express some glial and neuronal markers. In this study, we have sought to determine whether our observations represent actual expression of these markers or result from cross-reactivity. Immunohistochemistry was performed on human adult dermal fibroblasts using acknowledged glial and neuronal antibodies followed by verification of the data using RT-qPCR. Human adult dermal fibroblasts showed expression of the glia-specific markers SOX9, glial fibrillary acidic protein and EGR2 (KROX20) as well as for the neuron-specific marker class III β-tubulin, both at the protein and mRNA level. Furthermore, human adult dermal fibroblasts showed false-positive immunostaining for S100β and GAP43 and to a lower extent for OCT6. Our results indicate that immunophenotyping as a tool to determine cellular identity is not as reliable as generally assumed, especially since human adult dermal fibroblasts may be mistaken for neural cells, indicating that the ultimate proof of glial or neuronal identity can only be provided by their functionality.

## Introduction

The development of stem-cell-based therapies has been extensively explored during the past decade, with special emphasis on the use of pluripotent embryonic stem (ES) cells. Adult stem cells may represent a useful alternative source of donor cells, especially since ethical issues regarding human ES cells can be avoided. In addition, concomitant immunosuppressive therapy is likely to become irrelevant when treating patients with autologous stem cells. Furthermore, these cells can often be generated in large quantities. Adult autologous stem cells may be obtained from bone marrow, adipose tissue and skin, and therefore these alternative sources are already used in various clinical trials [1]. However, a major problem with such an approach is the undesirable heterogeneity of the harvested cell populations, depending on the tissue used. Fibroblasts are common constituents in most tissues and have high doubling rates, while culturing techniques in order to selectively remove fibroblasts—and, hence, to obtain a homogeneous stem cell population—often fail [2]. Therefore, it seems likely that fibroblast contamination is the reason for some unexplained discrepancies in clinical outcome after stem cell therapy [3].

Fibroblasts represent a phenotypically heterogeneous population of cells, which makes it difficult to identify and to eliminate them. Fibroblasts also display an immunophenotype similar to that of certain types of stem cells and can survive and proliferate during culture conditions used for a variety of (stem) cells [3], which may lead to misinterpretation of immunohistochemical data. Although several markers may be used for the proper identification of fibroblasts in tissue, it should be stressed that micro-environmental cues, elicited from isolation or cell culture procedures, can induce an altered cell phenotype resulting in *ex vivo* immunostaining patterns different from those *in vivo* [4, 5]. In general, fibroblasts are identified by their spindle-shaped morphology combined with immunostaining for the mesenchymal marker vimentin, whereas they do not demonstrate any immunostaining for markers specific for other mesenchymal cell types—such as muscle cells, astrocytes and hematopoietic cells—or epithelial markers [6]. However, under *in vitro* conditions various cell types may change towards a more migratory phenotype and they consequently will show features of a temporary epithelial-mesenchymal transition, including upregulation of vimentin [7–9]. Moreover, stem cells can also express vimentin [10]. This phenomenon makes it extremely difficult to distinguish between cultured, migrating (stem) cells and fibroblasts *in vitro*. Markers such as fibroblast surface antigen, CD34 or HSP47 are not sufficiently specific, because they are also expressed in other mesenchymal cells. Procollagen 1a is a reliable fibroblast marker, but its expression depends on the presence and level of TGF-β, and only active fibroblasts will produce collagen. It has been shown that the TE-7 antibody specifically recognizes growing and quiescent cultured fibroblasts in acetone- and formalin-fixed tissues and cells [11, 12]. However, Pilling et al. [13] have shown that the TE-7 antigen is inconsistently expressed, indicating that it is not a reliable marker.

Alternatively, the degree of fibroblast contamination can be determined indirectly by identification and establishment of the percentage of true stem cells and their progeny. It is generally acknowledged that many stem cell and progenitor cell antibodies are cell-specific and this, therefore, could give more certainty about the homogeneity of the stem cell population.

Our research program focuses on the development of a cell-replacement therapy to repair the auditory nerve using autologous stem cells. For that purpose, we isolate neural-crest-derived stem cells from the hair follicle bulge and investigate their potential to differentiate into glial cells and neurons [14]. Using fibroblasts from a primary culture of human adult dermal fibroblasts (HDF-a) as negative control, we frequently observe distinct immunostaining for class III β-tubulin (TUBB3), a commonly accepted marker for neurons. This observation prompted us to investigate if antibodies of recognized specificity against glial and neuronal proteins show positive immunostaining in HDF-a and, if so, whether this is supported by gene expression or due to a false-positive result. In this study, expression of glial and neuronal markers in HDF-a and human skin sections was analysed using immunostaining with a number of acknowledged neuronal and glial antibodies and quantitative reverse transcription (RT-qPCR).

## Materials and Methods

### Cell cultures

Primary human adult dermal fibroblasts (HDF-a, catalog number 2320, ScienCell, Carlsbad, CA, USA) were cultured in DMEM/F12 (Biochrom, Berlin, Germany) containing 10% fetal bovine serum (FBS, Life Technologies, Carlsbad, CA, USA), GlutaMAX (2mM L-alanyl-L-glutamine, Life Technologies) and 1% antibiotic antimycotic solution Sigma-Aldrich, Saint-Louis, MO, USA). The rat schwannoma cell-line (RT4-D6P2T, catalog number CRL-2768, ATCC, Manassas, VA, USA) was cultured in high-glucose DMEM (Life Technologies) with 10% FBS. Human astrocytes (HA, catalog number 1800, ScienCell) were cultured in DMEM/F12 containing 10% FBS to which were added 1% N-2 MAX Media Supplement R&D Systems, Minneapolis, MN, USA) and 20 ng/mL recombinant human EGF (R&D Systems). All cell lines were cultured in a humidified incubator with 5% CO_2_ at 37°C and medium was changed every other day. When cells reached 80% confluency, the cells were passaged using 0.05% trypsin/EDTA in PBS (Life Technologies). At passage 5, the cells were fixed in 1% formaldehyde in PBS (15 minutes), washed with PBS and processed for immunohistochemistry or lysed in RNA-Bee and stored at -20°C for RNA isolation.

### Tissue sample collection, fixation and cryo-sectioning

The human material was obtained from vestibular schwannoma (VS) during surgery and skin biopsies, as part of routine histopathological examination. All human material was handled according to the Dutch Medical Treatment Agreement Act (Dutch Civil Code, Book 7, Section 7.7.5, article 7:467; http://www.dutchcivillaw.com/legislation/dcctitle7777.htm). Patients gave informed consent. Samples from VS connected to cochleovestibular nerve were immediately frozen on dry ice and stored at -20°C for RNA isolation and RT-qPCR.

Sciatic nerve samples were obtained from surplus mice. The care and use of the animals were according to the regulations as stipulated by the Dutch Experiments on Animals Act (WoD) and the European Directive on the Protection of Animals Used for Scientific Purposes (2010/63/EU). Experimental procedures were approved by the Central Animal Ethics Committee and the LUMC Animal Welfare Body (DEC permit 10172). Samples were fixed overnight in 4% formaldehyde in PBS at 4°C. Fixed tissues were washed in PBS and embedded in Tissue-Tek^®^ O.C.T.^™^ compound (Sakura, Alphen a/d Rijn, the Netherlands), rapidly frozen in liquid nitrogen and stored at -20°C. Sections (10 μm) were cut in a cryostat microtome at a -20°C, thaw-mounted onto gelatin-coated slides, fixed in ice-cold acetone (10 minutes), and subsequently air-dried.

### Immunohistochemistry

The samples, both cells and sections, were washed for 5 minutes in 0.05% Tween-20 in PBS. Samples, except those for p75^NTR^ immunostaining, were permeabilized with 0.1% Triton X-100 in PBS (10 minutes), washed in 0.05% Tween-20 in PBS (5 minutes) and blocked (10 minutes) with blocking buffer consisting of 5% non-immune serum in 0.05% Tween-20 in PBS. Subsequently, samples were immunostained for markers specific for neural crest cells (nestin, SOX9, SOX10, and p75^NTR^) [15], glial cells (S100β, GFAP, OCT6, EGR2) [16, 17], GAP43, a marker for both, glial cells and neurons [18, 19] and neuronal cells (TUBB3, NEFM, and NEFH) [20]. Primary antibodies are listed in Table 1. Antibodies were diluted in blocking buffer and samples were incubated overnight at 4°C. Subsequently, samples were washed in PBS for three times, blocked and incubated with the secondary antibodies diluted in blocking buffer (Table 2) for 1 hour at room temperature followed by three washes with PBS. Cell nuclei were stained with 5 μg/ml DAPI in Vectashield (Sigma-Aldrich). The samples were then covered with cover glasses.

**Table 1:** Primary antibodies used in this study.

*Primary antibodies*	Dilution	Company	Catalog number	Positive control
Class III β-tubulin (TUJ1, rabbit monoclonal)	1:500	Covance, Princeton, NJ, USA	MRB-435P	Mouse sciatic nerve
Class III β-tubulin (2G10, mouse monoclonal)	1:200	Abcam, Cambridge, UK	ab78078	Mouse sciatic nerve
Class III β-tubulin (TUBB3, rabbit polyclonal)	1:200	Abcam, Cambridge, UK	ab18207	Mouse sciatic nerve
Growth associated protein 43 (GAP43, rabbit polyclonal)	1:500	Novus Biologicals, Ontario, Canada	NB300-143	RT4-D6P2T
Glial fibrillary acidic protein (GFAP, rabbit polyclonal)	1:500	DAKO, Glostrup, Denmark	Z0334	RT4-D6P2T
Glial fibrillary acidic protein (GFAP, mouse monoclonal)	1:100	Abcam, Cambridge, UK	ab10062	Human astrocytes
EGR2 (rabbit polyclonal)	1:100	Covance, Princeton, NJ, USA	PRB-236P	RT4-D6P2T
Nestin (clone 4D11, mouse monoclonal)	1:200	Biosensis, Thebarton, Australia	M-1385-100	RT4-D6P2T
Neurofilament heavy chain (NEFH, mouse monoclonal)	1:200	Abcam, Cambridge, UK	Ab19386	Mouse sciatic nerve
Neurofilament medium chain (NEFM, mouse monoclonal)	1:200	DSHB, Iowa City, IA, USA	2H3	Mouse sciatic nerve
OCT6 (POU3f, goat polyclonal)	1:50	Santa-Cruz Biotechnology, Dallas, TX, USA	sc-11661	RT4-D6P2T
p75^NTR^ (rabbit polyclonal)	1:200	Merck Millipore, Darmstadt, Germany	07–476	RT4-D6P2T
S100β (mouse monoclonal)	1:500	Sigma-Aldrich, Saint-Louis, MO, USA	S2532	RT4-D6P2T
S100β (rabbit monoclonal)	1:200	Abcam, Cambridge, UK	ab52642	RT4-D6P2T
SOX9 (rabbit polyclonal)	1:200	Merck Millipore, Darmstadt, Germany	AB5535	RT4-D6P2T
SOX10 (goat polyclonal)	1:50	Santa-Cruz Biotechnology, Dallas, TX, USA	sc-17342	RT4-D6P2T

**Table 2:** Secondary antibodies used in this study.

*Secondary antibodies*			
Goat anti-rabbit IgG/Alexa Fluor 555 conjugate	1:200	Life Technologies Carlsbad, CA, USA	A21428
Goat anti-mouse IgG/Alexa Fluor 488 conjugate	1:200	Life Technologies Carlsbad, CA, USA	A11001
Donkey anti-goat IgG/Alexa Fluor 488 conjugate	1:200	Life Technologies Carlsbad, CA, USA	A11055

Since positive control tissues from human origin were not sufficiently available, a human astrocyte cell line (ScienCell) and a rat schwannoma cell line (ATCC) as well as mouse sciatic nerve were used as positive controls for immunostaining (Table 1). Cross-reactivity of the secondary antibody was ruled out by omitting the primary antibody in a parallel set of incubations.

### Fluorescence microscopy

For each of the markers studied, fluorescence microscopy was applied for HDF-a, human skin sections and the respective positive and negative controls. Images from human skin sections have been taken from those areas where some staining was visible. In general, the dermal part of skin sections have been shown, because staining—when present—was in the dermal part of the skin.

Digital images were obtained with an Olympus IX70 fluorescence microscope (Olympus, Tokyo, Japan) equipped with a Leica DFC340 FX camera using Leica Application Suite Advanced Fluorescence software (LAS AF). All images were acquired within simultaneous series and digitally stored using the same settings.

### Quantitative reverse transcription PCR analysis

Primers were designed with Primer-BLAST (http://www.ncbi.nlm.nih.gov/tools/primer-blast). Total RNA was isolated from human astrocytes and HDF-a cells at passage 5 and human VS tissue using RNA-Bee (Tel-Test Inc., Friendswood, TX, USA) according to the manufacturer’s instructions. cDNA was generated by incubating 1 μg RNA with 500 ng Random Hexamers (Promega, Madison, WI, USA) supplemented with H_2_O to a final volume of 10 μl, for 10 minutes at 70°C. To each sample a mix of 100 units M-MLV reverse transcriptase, 1.25 μl 10 mM dNTP mix, 20 units RNAsin^®^ RNase inhibitor and 1x M-MLV Reverse Transcriptase buffer (all from Promega) was added in a total volume of 10 μl, incubated at room temperature for 10 minutes, and heated for 50 minutes at 45°C and 10 minutes at 70°C. RT-qPCR was performed using IQ^™^Sybr^®^ Green Supermix (Bio-Rad, Hercules, CA, USA) in a CFX96 Touch Real-Time PCR Detection System (Bio-Rad) with an initial denaturation step of 3 minutes at 95°C followed by 40 cycles of 10 seconds at 95°C and 30 seconds at 60–62°C, the latter depending on the primer set used. Cycle threshold values were attained and relative expression levels of mRNA were normalized to the housekeeping gene β2-microglobulin and calculated by the 2^-ΔΔCt^ method [21]. PCRs were performed in triplicate for each primer set. All primers used with the appropriate annealing/extension temperatures are listed in Table 3. Values represent mean ± SD. Statistical analysis using an unpaired two-tailed Student’s T-test was performed using Graphpad Prism 5 software (La Jolla, CA, USA). Differences were considered significant at p < 0.05.

**Table 3:** List of primers used in RT-qPCR.

Gene	Forward primer	Reverse primer	Annealing/extension temperature
*β2-microglobulin*	TGC-TGT-CTC-CAT-GTT-TGA-TGT-ATC-T	TCT-CTG-CTC-CCC-ACC-TCT-AAG-T	60
*Nestin*	CTG-CGG-GCT-ACT-GAA-AAG-TTC	CTG-AGC-GAT-CTG-GCT-CTG-TA	60
*p75* ^*NTR*^	CGA-CAA-CCT-CAT-CCC-TGT-CT	GTT-GGC-TCC-TTG-CTT-GTT-CTG	62
*SOX9*	GTA-CCC-GCA-CTT-GCA-CAA-C	CAC-CGA-CTT-CCT-CCG-CC	62
*SOX10*	ATG-TCA-GAT-GGG-AAC-CCC-GA	TGG-ACA-TTA-CCT-CGT-GGC-TG	58
*S100β*	TCT-GGA-AGG-GAG-GGA-GAC-AA	GGA-AGT-CAC-ATT-CGC-CGT-CT	62
*GFAP*	TGT-CAG-AAG-GCC-ACC-TCA-AG	AGA-GGC-GGA-GCA-ACT-ATC-CT	62
*OCT6/POU3f*	GCT-CGA-GAG-CCA-CTT-TCT-CA	GTC-ATG-CGC-TTC-TCC-TTC-TG	62
*EGR2*	GAC-CAT-CTT-TCC-CAA-TGC-CG	TTT-CTA-GGT-GCA-GAG-ACG-GG	62
*GAP43*	CAA-CCA-TGC-TGT-GCT-GTA-TGA-G	GTG-TTA-TGA-CCT-CGT-CAC-CCA	60
*TUBB3*	GAG-GGA-GAT-CGT-GCA-CAT-CCA-GG	CGA-GTC-GCC-CAC-GTA-GTT-GC	62
*NEFM*	CCA-AGG-AAG-AGA-TCG-CCG-AG	GTG-CCC-CGA-AGC-TCA-TTT-TC	62
*NEFH*	GAG-GAA-CAC-CAA-GTG-GGA-GA	AAG-CGA-GAA-AGG-AAT-TGG-GC	60

Primers were used with the appropriate annealing temperature for RT-qPCR.

## Results

### Immunohistochemistry

**Fig 1:**
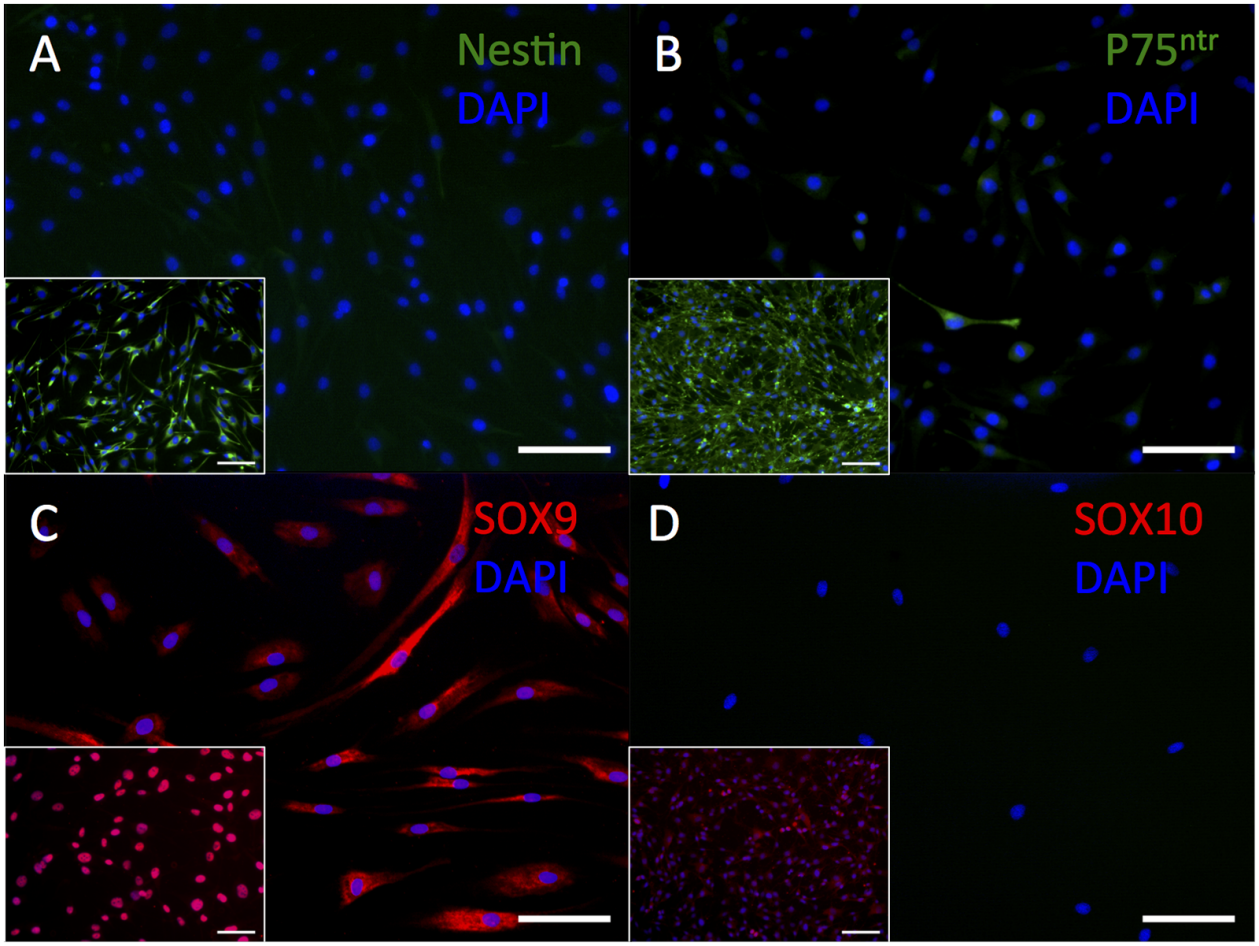
Immunostaining of HDF-a for nestin, p75^NTR^, SOX9 and SOX10. Human adult dermal fibroblasts do not immunostain for nestin (A,) but very few cells immunostain faintly for p75^NTR^ (B). Human adult dermal fibroblasts show positive immunostaining for the nuclear marker SOX9 (C), but not for SOX10 (D). Inserts: rat schwannoma cell line as positive control. Cell nuclei are stained blue with DAPI. Scale bar = 100 μm.

HDF-a did not show immunostaining for the neural stem cell marker nestin (Fig 1A). The low-affinity nerve growth factor receptor p75^NTR^, which is expressed during all phases of Schwann cell development except in myelinating Schwann cells, was stained in very few cells, both in the cytoplasm and at the cell surface (Fig 1B), contrary to rat schwannoma cells (positive control) showing bright fluorescence only at their cell membranes. HDF-a showed cytoplasmic immunostaining for the neural-crest and immature Schwann cell marker SOX9 (Fig 1C), although it is a nuclear marker, as shown in the rat schwannoma cell line. SOX10 which is expressed at all developmental stages of Schwann cells [22], could not be detected in HDF-a (Fig 1D).

**Fig 2:**
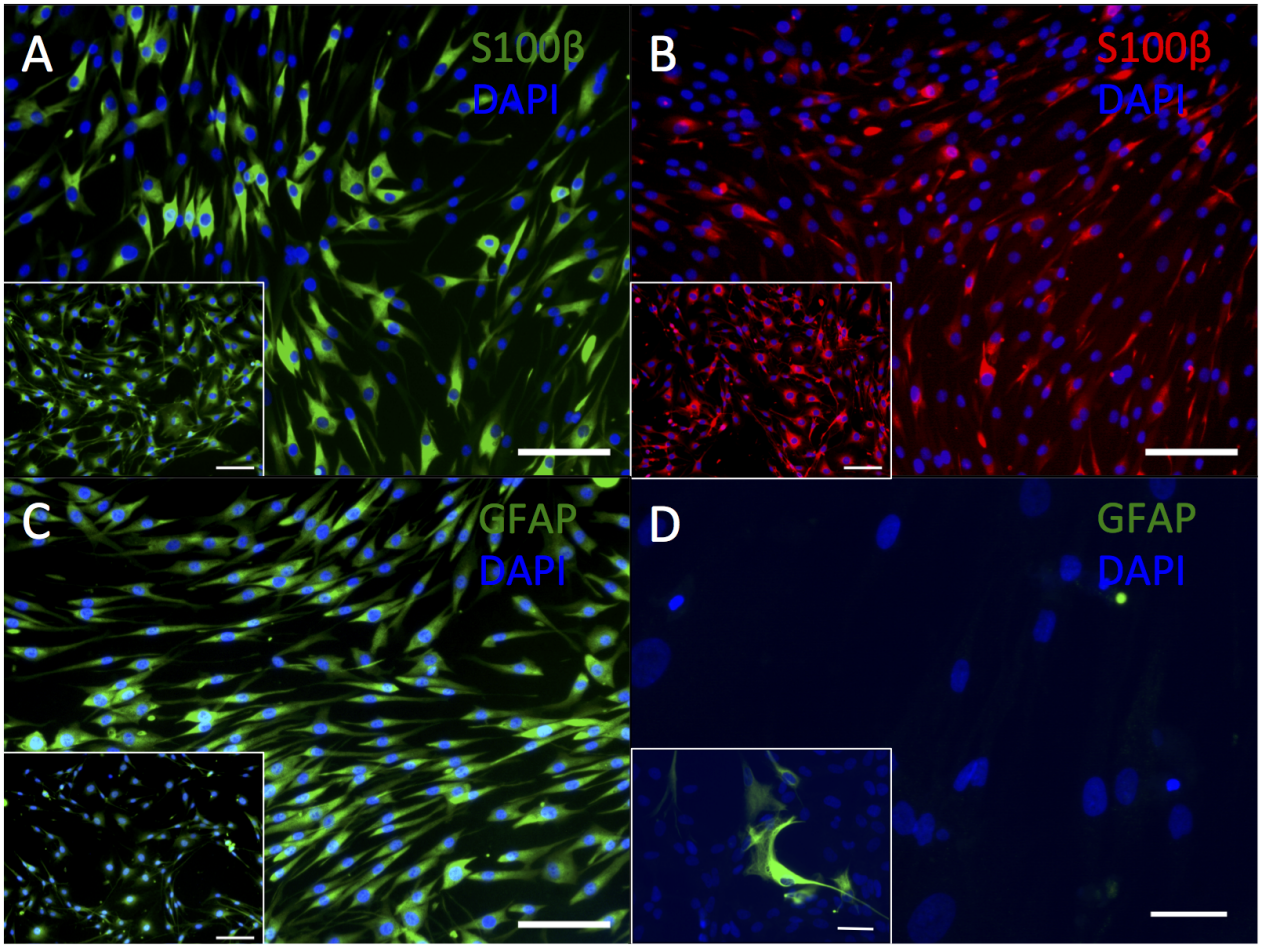
Immunostaining of HDF-a for S100β and GFAP. Human adult dermal fibroblasts express S100β Sigma (A), S100β Abcam (B) and polyclonal GFAP (DAKO; C) but do not immunostain for monoclonal GFAP (Abcam; D). Inserts: rat schwannoma cell line (A-C) and human astrocytes (D) as positive controls. Cell nuclei are stained blue with DAPI. Scale bar = 100 μm (A- C) and 50 μm (D).

**Fig 3:**
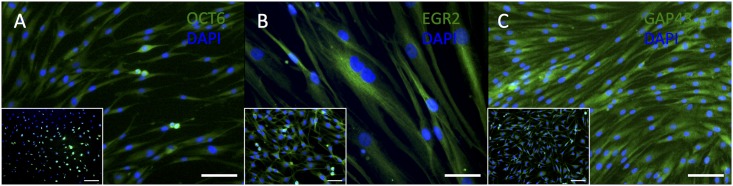
Immunostaining of HDF-a for OCT6, EGR2 and GAP43. Human adult dermal fibroblasts do not express OCT6 (A) and express EGR2 and GAP43 (B,C). Inserts: rat schwannoma cell line (A-C) as positive controls. Cell nuclei are stained blue with DAPI. Scale bar = 100 μm (A, C) and 50 μm (B).

A predominantly cytoplasmic immunostaining for S100β (using antibodies from Sigma and Abcam, Fig 2A and 2B) was observed in HDF-a, similar to the immunostaining pattern observed in the rat schwannoma cell line. Cytoplasmic immunostaining for GFAP (Fig 2C) was clearly visible in HDF-a using the polyclonal antibody from DAKO, but was absent if the monoclonal antibody from Abcam was applied (Fig 2D). The expression pattern of GFAP from DAKO in HDF-a corresponded to that of the positive control. OCT6, which is required in immature Schwann cells for their differentiation into myelinating cells, was weakly expressed in the cytoplasm of HDF-a (Fig 3A), most probably aspecific binding of the antibody. EGR2 –present in mature, myelinating, Schwann cells [17]–showed a predominantly cytoplasmic immunostaining in HDF-a (Fig 3B), similar to the positive control. GAP43 was expressed in Schwann cell precursors as well as in immature and non-myelinating Schwann cells [17], as well as in some types of neurons [19], but is also present in the cytoplasm of HDF-a (Fig 3C).

**Fig 4:**
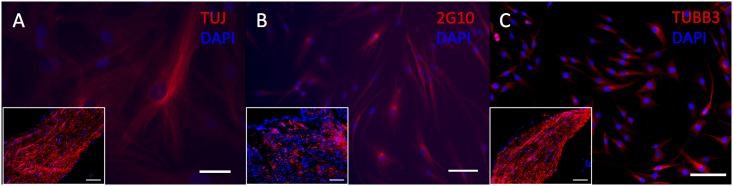
Immunostaining of HDF-a for TUBB3. Human adult dermal fibroblasts express clone TUJ (A), 2G10 (B) and polyclonal TUBB3 (C). Inserts: mouse sciatic nerve as positive control. Cell nuclei are stained blue with DAPI. Scale bar = 50 μm (A) and 100 μm (B-C).

**Fig 5:**
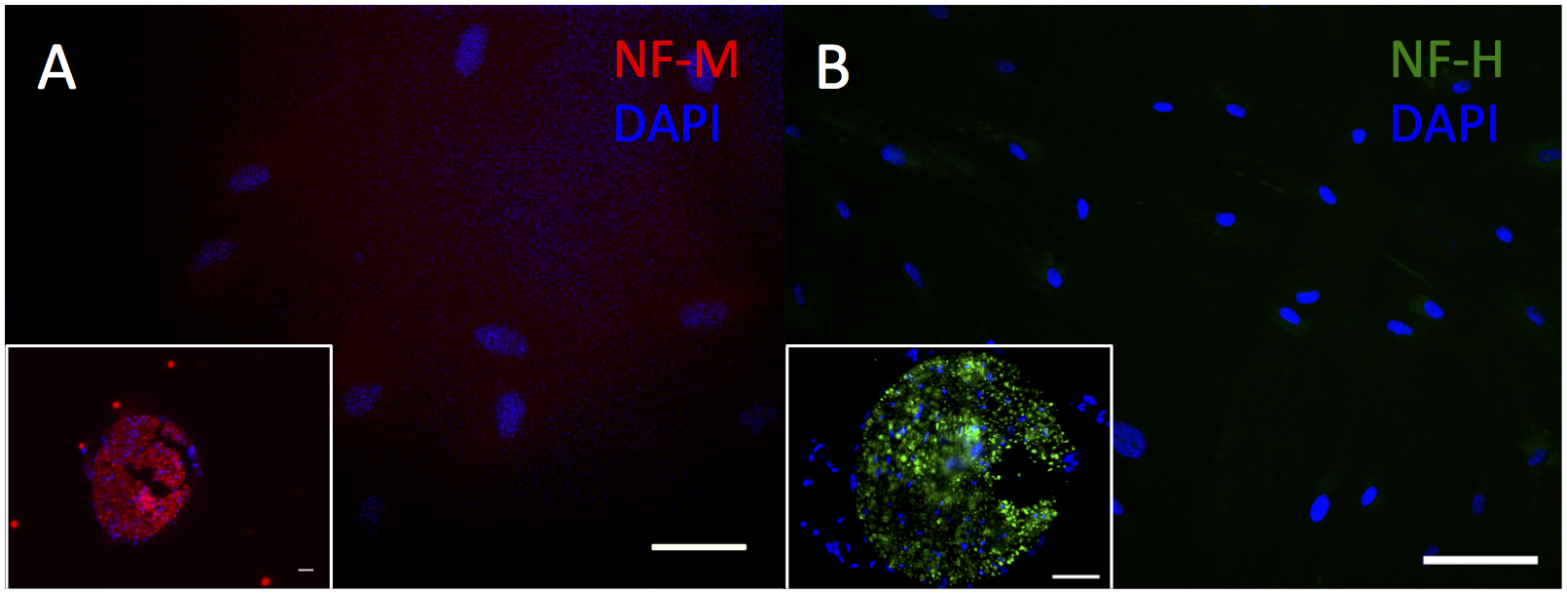
Immunostaining of HDF-a for NEFM and NEFH. Human adult dermal fibroblasts do not immunostain for NEFM (A) and NEFH (B). Inserts: mouse sciatic nerve (A-B), used as positive control. Cell nuclei are stained blue with DAPI. Scale bar = 50 μm (A) and 100 μm (B).

Cytoplasmic immunostaining was observed for all TUBB3 antibodies (TUJ1, Covance; 2G10, Abcam; TUBB3, Abcam) in HDF-a and this corresponds to the expression pattern observed in mouse sciatic nerve (Fig 4A–4C). HDF-a did not demonstrate immunostaining for either NEFM or NEFH (Fig 5A–5B). In human skin, none of the used antibodies, showed a clear positive immunostaining (S1 to S5 Figs).

### Quantitative reverse transcription PCR analysis

**Fig 6:**
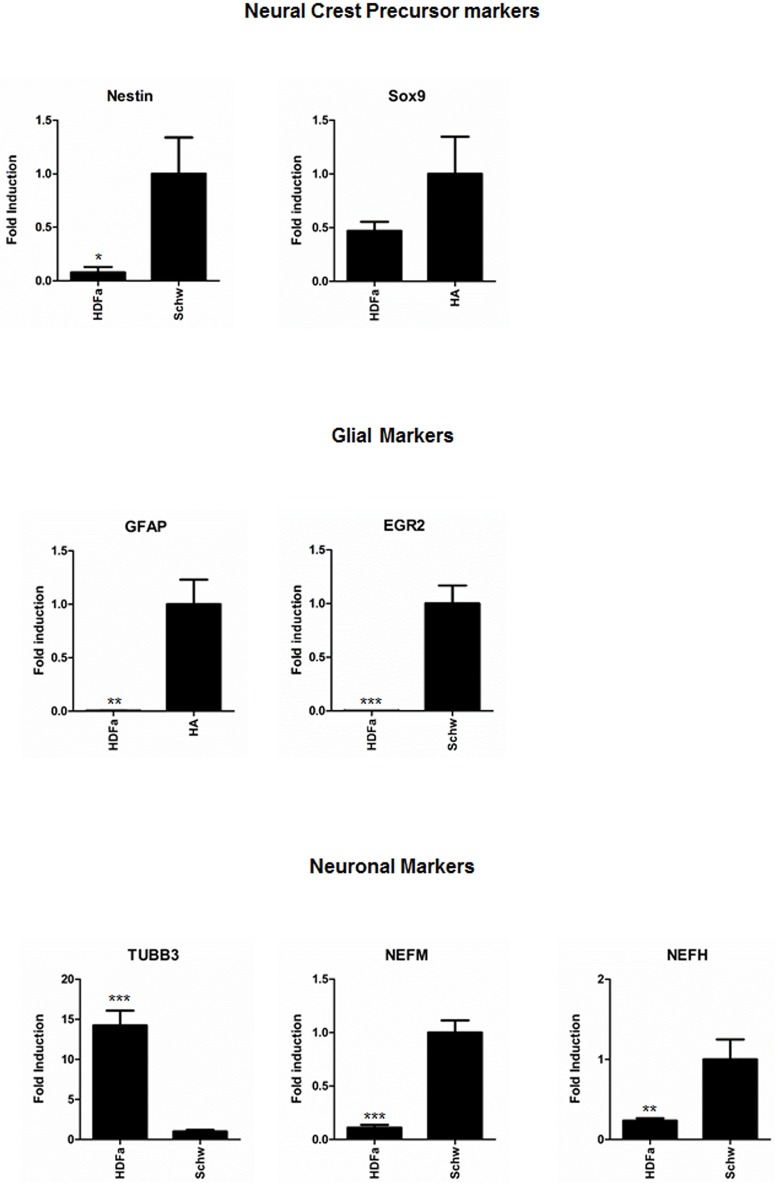
Quantitative reverse transcription PCR analysis of neural-crest, glial and neuronal markers in HDF-a compared to positive controls. RT-qPCR analysis was performed to determine the gene expression levels of neural-crest markers *nestin* and *SOX9* (top), glial markers *GFAP* and *EGR2* (middle) and neuronal markers *TUBB3*, *NEFM* and *NEFH* (bottom) in samples of HDF-a. Expression levels of neural-crest and glial markers were compared to expression in human astrocytes and human vestibular schwannoma respectively, using the 2^-ΔΔCt^ method, while expression of neuronal markers was compared to human vestibular schwannoma samples. HDF-a = human adult dermal fibroblasts; HA = human astrocytes; Schw = human vestibular schwannoma; *TUBB3* = class III β-tubulin; *NEFM* = neurofilament medium chain; *NEFH* = neurofilament heavy chain. * p<0.05; ** p<0.01; *** p<0.001.

To corroborate the immunostaining data, we performed real-time RT-qPCR analysis of all neural-crest, glial and neuronal markers in low-passage (passage 5) samples of HDF-a. Expression levels of neural-crest and glial markers in HDF-a were compared to those in human astrocytes and human vestibular schwannoma respectively, using the 2^-ΔΔCt^ method, and the expression levels of neuronal markers in HDF-a were compared to those in human vestibular schwannoma samples. *Nestin* was expressed in HDF-a, although the level of expression was approximately 12-fold lower (p<0.05) than in vestibular schwannoma (Fig 6, top left). The neural-crest markers *p75* ^*NTR*^, *SOX9* and *SOX10* were found to be differentially expressed. *SOX9* was clearly expressed at the gene level (Fig 6, top right), in contrast to *p75* ^*NTR*^ and *SOX10*. *SOX10* could not be detected, while expression of *p75* ^*NTR*^ was just detectable (data not shown).

*S100β* expression could not be detected by RT-qPCR in HDF-a (data not shown). Expression of *GFAP* was at detection threshold (Fig 6, middle). Furthermore, the glial marker *OCT6* and *GAP43*, marker for glial cells and neurons, could not be detected by RT-qPCR in HDF-a, although their expression was above detection level in the positive controls (data not shown). Gene expression of EGR2, a protein produced in Schwann cells at the onset of myelination, was present in HDF-a, albeit at low levels (Fig 6, middle). Finally, *TUBB3* expression in HDF-a was 14-fold higher (p<0.001) than in human vestibular schwannoma (Fig 6, bottom). Both *NEFM* and *NEFH* were expressed in HDF-a, but at low levels (p<0.01, Fig 6, bottom).

## Discussion

This study demonstrates that human adult dermal fibroblasts do express the glia-specific markers SOX9, GFAP and EGR2 as well as the neuron-specific marker TUBB3. The immunostaining data have been corroborated by gene expression analysis using RT-qPCR. Furthermore, these fibroblasts show false-positive immunostaining for S100β, GAP43 and, to a lower extent, OCT6. It is noteworthy, that in previous experiments in our laboratory, these antibodies did not show propensity for non-specific binding, because immunostaining on different cells, e.g., keratinocytes, yielded a negative result.

Immunophenotyping as a tool to determine cellular identity is not as reliable as generally assumed. In this paper we show that fibroblasts can display a neuroglial phenotype. This phenomenon will hamper the establishment of cellular identities of neuron-like and glia-like cells, especially those in stem cell populations, which by reason of their origin, may contain fibroblasts as well. The observation that fibroblasts express proteins, generally assumed to be solely present in glial cells and neurons is interesting from the point of view of their pleiotropism, but is worrisome concerning the interpretation of immunohistochemical observations. We have shown that parallel incubation with positive control samples are crucial for comparison of expression levels (p75^NTR^ and OCT6) and cellular immunostaining patterns (SOX9, OCT6 and EGR2). However, not all immunostainings in human fibroblasts might be due to cross-reactivity, for RT-qPCR also showed expression at the gene level of *SOX9*, *GFAP*, *EGR2*, and *TUBB3*. Another issue is the finding that the immunostaining data for GFAP are partially different from the RT-qPCR results. HDF-a cells were found immunoreactive for the polyclonal GFAP antibody (DAKO), but not for the monoclonal GFAP antibody (Abcam). This discrepancy can be explained by the fact that polyclonal antibodies recognize multiple epitopes of the same antigen and that a monoclonal antibody generally displays a low avidity for its epitope on the antigen.

The RT-qPCR results also show that fibroblasts express *nestin*, although in our hands, this could not be corroborated with immunohistochemistry, which might be due to posttranslational mRNA or protein degradation [23]. Under normal circumstances, *SOX9* and *TUBB3* mRNA are not expressed in human dermal fibroblasts [24]. To the best of our knowledge, expression patterns of nestin, EGR2 or GFAP on the gene and protein level have not been investigated before in normal human dermal fibroblasts.

The aforementioned gene expression in fibroblasts might be a sign of cellular stress or may represent a more or less continuous proliferative phase of cells, which is common in most cell cultures. This theory is supported by the faint immunostainings in the human skin tissue sections, considering that normal skin represents a quiescent *in vivo* situation. In our opinion, the expression of S100β in mouse embryonic fibroblast cell lines fits well with this theory [25]. Elaborating on this, SOX9 expression is often nuclear, however, depending on the cell type and cellular circumstances, cytoplasmic expression has been reported [26, 27]. The cytoplasmic presence of SOX9 in NIH3T3 fibroblasts and in invasive tumor cells shows involvement of SOX9 in epithelial mesenchymal transition, i.e. cells with a migratory, proliferative cellular phenotype. This phenomenom has been described as a permanent character in tumors and of a reversible nature in wound healing [28]. The latter may also hold true for cells in culture for the effect caused by culture circumstances [29]. The cytoplasmic expression of SOX9 in HDF-a may therefore be the result of temporary upregulation, due to epithelial mesenchymal transition, based on both the cell type (fibroblasts) and culture circumstances. This is reflected in the RT-qPCR values.

Cell culture environment might not only induce stress- or proliferation-related protein expression. Cells may also change into an activated state resembling that of a wound-healing mechanism. For instance, it has been reported that portal fibroblasts express neural cell adhesion molecule (NCAM) during liver regeneration in adult rats after partial hepatectomy [16]. This study corroborates that fibroblast NCAM expression in regenerating tissue is induced by cellular activation [30]. Cell migration is essential in stress, proliferation and wound healing. Stress-activated cells will migrate to seek contact with congeners, proliferative cells have to seek a vacant habitat and, in wound healing, cells have to migrate to the site of the wound. To serve migration, the cell has to re-arrange protein functions, while many other proteins that are uncommon in quiescent cells, such as actomyosin, will have to be produced [31, 32]. The phenomenon that cell activation and migration induce pleiotropism may also explain why TUBB3 is present in other cells, such as melanocytes [33]. Although a dynamic role for TUBB3 in multiple aspects of melanocyte function has been suggested (i.e., the onset of melanogenesis), we have also observed its presence in hair follicle medulla keratinocytes *in vivo* [33]. Expression of TUBB3 in the mitotic spindle of cultured keratinocytes and fibroblasts has been previously reported [34]. However, in this study we show that TUBB3 is also clearly expressed in non-dividing fibroblasts. Taken together, these findings suggest that protein pleiotropism for TUBB3, especially in cells derived from the skin. Therefore, proof of a neuron’s identity by means of immunostaining for TUBB3 alone should be rejected. This holds true for the identification of glial cells after glial induction as well. To overcome the difficulties of false interpretation of immunohistochemical data, we recommend to use parallel incubations with a pertinent cell or tissue control as well as a fibroblast culture (not a cell line) as a negative control and most importantly, a panel of antibodies as a selective tool prior to functionality studies.

Our results may have a major impact on protocols that are currently in use to identify stem cells harvested from a wide range of adult tissues, in particular those in which fibroblasts are ubiquitously present, such as adipose tissue, skin and bone marrow. Moreover, it has been suggested that cultured and expanded mesenchymal stem cells and fibroblasts are indistinguishable on basis of their morphology, cell surface markers, differentiation potential and immunologic properties [35]. Fibroblast function may differ throughout the body, but the essential function of all fibroblasts is the same: production of extracellular matrix [36]. Regarding the development of stem-cell-based therapies, it has to be kept in mind that, although the immunomodulatory properties from fibroblasts (like that of mesenchymal stem cells) might be an important feature after cell transplantation, their fibrogenic potential could well hinder the beneficial therapeutical effect. This has clearly been demonstrated in the study of Grigoriadis et al. [23], in which extracellular matrix was found in the brain after transplantation of bone marrow stem cells in a mouse model for autoimmune encephalomyelitis [37]. Recently, a group of internationally respected investigators has warned against the use of stem cells for therapeutic purposes without extensive exploration of their true nature, *in vitro* and *in vivo* [38]. Our study presents further substantiation of this warning, especially with regard to stem cell populations that may contain contaminating fibroblasts. In any case, the ultimate proof of neuronal and glial identity remains their functionality.

## Supporting Information

S1 Fig Immunostaining of nestin, p75^NTR^, SOX9 and SOX10 in human skin tissue sections. Cells in the dermis do not demonstrate immunostaining for nestin (A) or SOX10 (D). Faint immunostaining close to the epidermis is present for p75^NTR^ (B) in some cells, while SOX9 (C) expression is demonstrated by only a few cells in the dermis. Inserts: human adult dermal fibroblasts show absence of immunostaining for nestin(A) and SOX10 (D). Positive immunostaining for p75^NTR^ (B) is limited to some fibroblasts, whereas immunostaining for SOX9 (C) is present in all fibroblasts. Cell nuclei are stained blue with DAPI. Scale bar = 100 μm (A-D).
(TIF)

S2 Fig Immunostaining for S100β and GFAP in human skin tissue sections. Sections immunostained for S100β show either a faint background staining (A: Sigma antibody) or positive immunostaining of single cells (B: Abcam antibody). Immunostaining with the polyclonal GFAP antibody (C) is absent from the dermis, while the monoclonal GFAP antibody (D) shows weak staining in cells that form clusters. Inserts: Human dermal fibroblasts immunostain for both S100β antibodies (A: Sigma; B: Abcam) as well as the polyclonal GFAP antibody (C), but do not react with the monoclonal GFAP antibody (D). Cell nuclei are stained blue with DAPI. Scale bar = 100 μm (A-B) and 50 μm (C-D).
(TIF)

S3 Fig Immunostaining for OCT6, EGR2 and GAP43 in human skin tissue sections. Nearly all cells in the dermis are negative for OCT6 (A), EGR2 (B) and GAP43 (C), although some single cells show faint immunostaining. Inserts: Human adult dermal fibroblasts do not immunostain for OCT6 (A), but are positive for EGR2 (B) and GAP43 (C). Cell nuclei are stained blue with DAPI. Scale bar = 100 μm (A-C).
(TIF)

S4 Fig Immunostaining for class III β-tubulin in human skin tissue sections. Some of the cells in the human skin tissue sections faintly react with either one of the antibodies used (A: TUJ; B: 2G10); no staining was visible for TUBB3 (C). Although some cells do seem to be faintly positive, the degree of immunostaining is much lower than that of human dermal fibroblasts (cf., inserts) and the positive control, i.e. sciatic nerve (see Results section). Inserts: Human dermal fibroblasts react with the monoclonal antibodies TUJ (A) and 2G10 (B) as well as the polyclonal TUBB3 antibody (C). Cell nuclei are stained blue with DAPI. Scale bar = 100 μm (A-C).
(TIF)

S5 Fig Immunostaining for NEFM and NEFH in human skin tissue sections. Cells in the dermis are negative for NEFM (A), although NEFH (B) immunostaining results in some faintly positive cells located beneath the epidermis, which may represent intradermal nerve endings. Inserts: Human dermal fibroblasts are negative for both NEFM (A) and NEFH (B). Cell nuclei are stained blue with DAPI. Scale bar = 50 μm (A) and 100 μm (B).
(TIF)
