# Supplementary figures and images for: Human Dermal Fibroblasts Demonstrate Positive Immunostaining for Neuron- and Glia- Specific Proteins
Source: PLoS One. 2015 Dec 17;10(12):e0145235. doi: 10.1371/journal.pone.0145235 (PMC4683011; doi:10.1371/journal.pone.0145235)

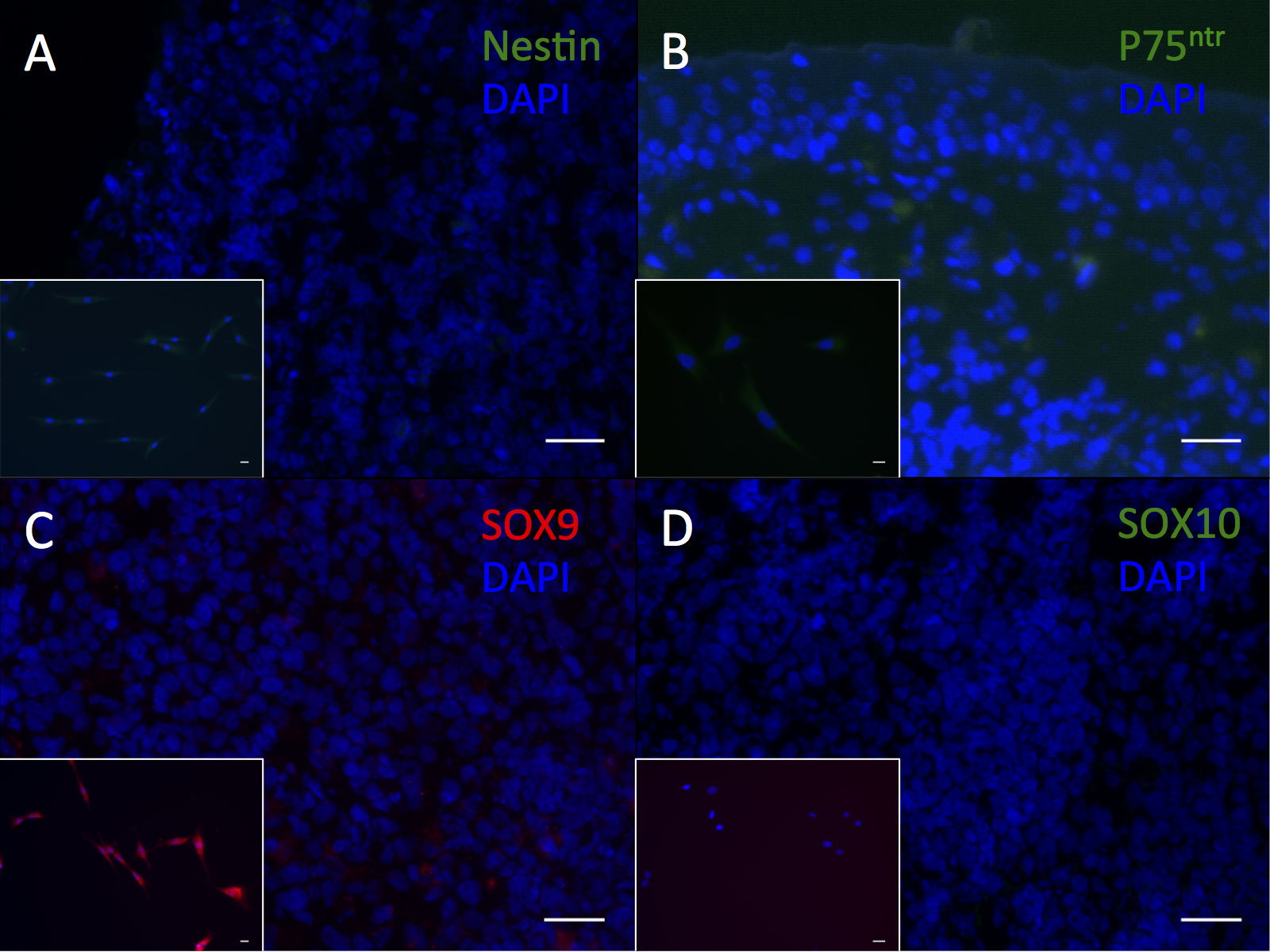

Supplement: S1 Fig — Cells in the dermis do not demonstrate immunostaining for nestin (A) or SOX10 (D). Faint immunostaining close to the epidermis is present for p75NTR (B) in some cells, while SOX9 (C) expression is demonstrated by only a few cells in the dermis. Inserts: human adult dermal fibroblasts show absence of immunostaining for nestin(A) and SOX10 (D). Positive immunostaining for p75NTR (B) is limited to some fibroblasts, whereas immunostaining for SOX9 (C) is present in all fibroblasts. Cell nuclei are stained blue with DAPI. Scale bar = 100 μm (A-D). (TIF) [file pone.0145235.s001.tif]

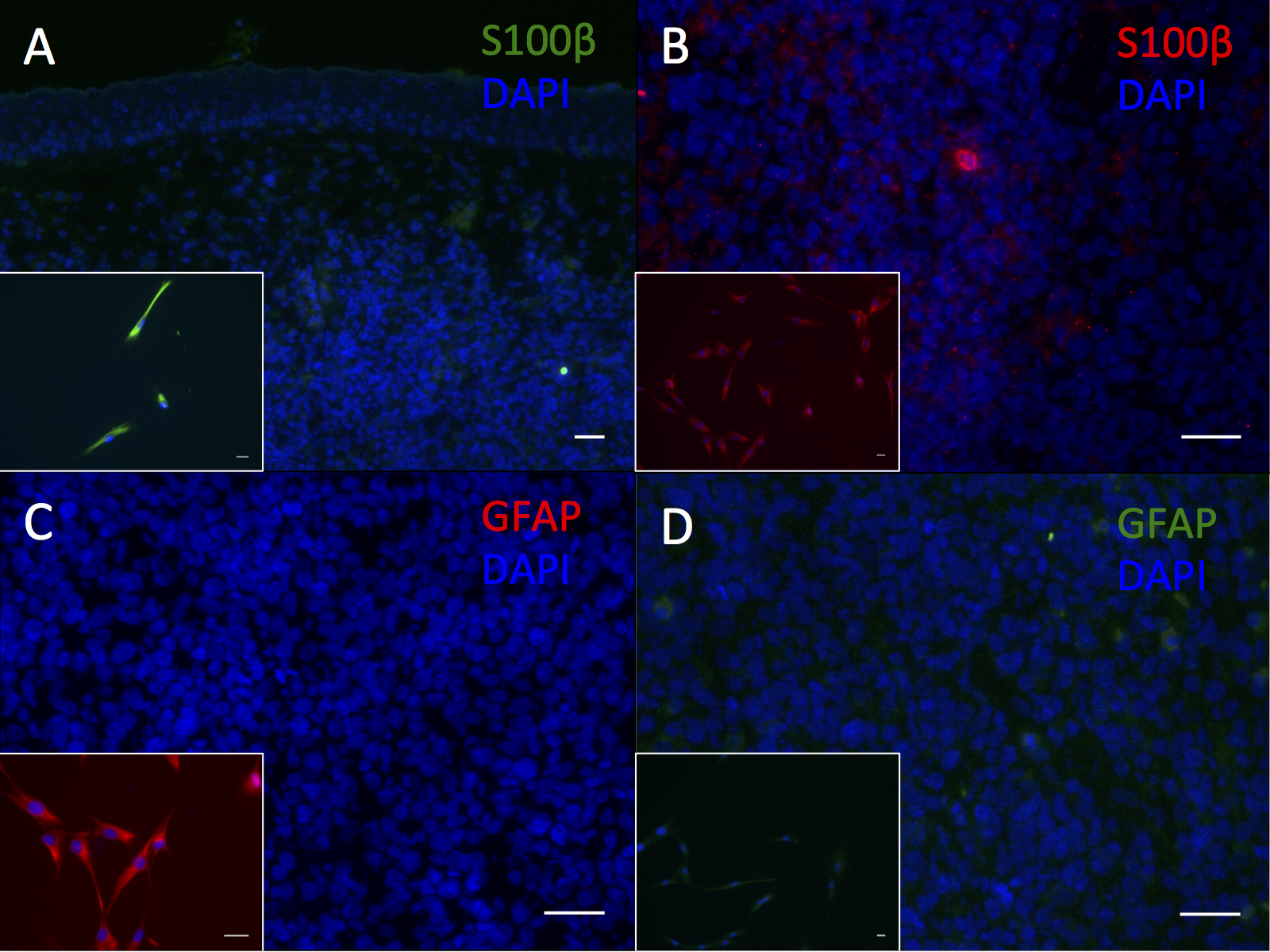

Supplement: S2 Fig — Sections immunostained for S100β show either a faint background staining (A: Sigma antibody) or positive immunostaining of single cells (B: Abcam antibody). Immunostaining with the polyclonal GFAP antibody (C) is absent from the dermis, while the monoclonal GFAP antibody (D) shows weak staining in cells that form clusters. Inserts: Human dermal fibroblasts immunostain for both S100β antibodies (A: Sigma; B: Abcam) as well as the polyclonal GFAP antibody (C), but do not react with the monoclonal GFAP antibody (D). Cell nuclei are stained blue with DAPI. Scale bar = 100 μm (A-B) and 50 μm (C-D). (TIF) [file pone.0145235.s002.tif]

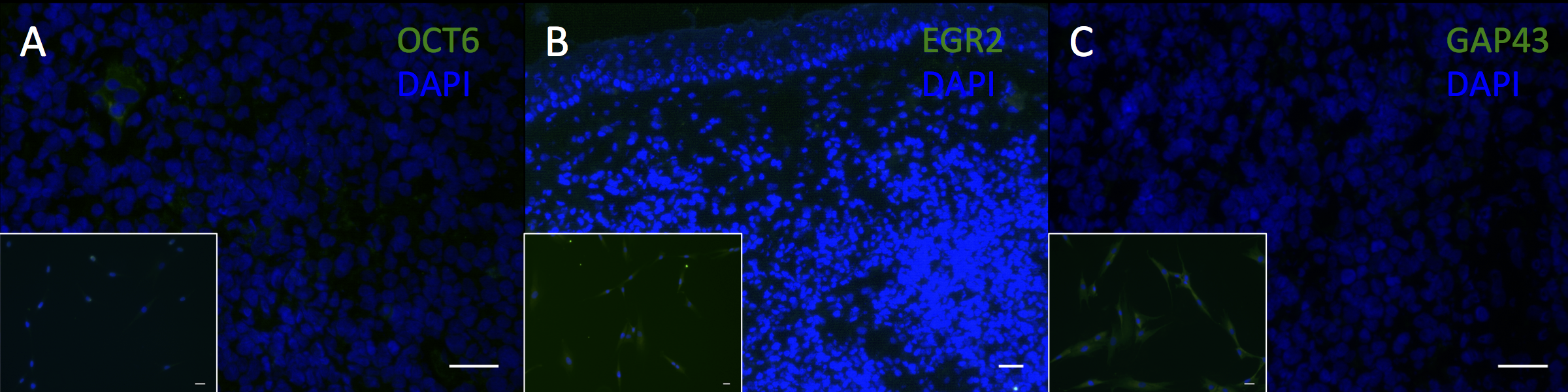

Supplement: S3 Fig — Nearly all cells in the dermis are negative for OCT6 (A), EGR2 (B) and GAP43 (C), although some single cells show faint immunostaining. Inserts: Human adult dermal fibroblasts do not immunostain for OCT6 (A), but are positive for EGR2 (B) and GAP43 (C). Cell nuclei are stained blue with DAPI. Scale bar = 100 μm (A-C). (TIF) [file pone.0145235.s003.tif]

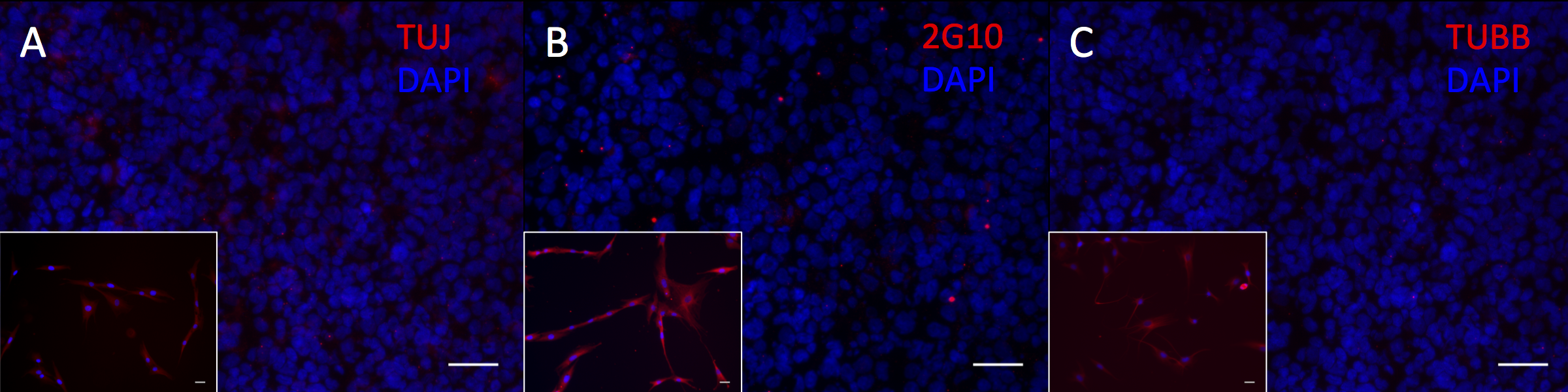

Supplement: S4 Fig — Some of the cells in the human skin tissue sections faintly react with either one of the antibodies used (A: TUJ; B: 2G10); no staining was visible for TUBB3 (C). Although some cells do seem to be faintly positive, the degree of immunostaining is much lower than that of human dermal fibroblasts (cf., inserts) and the positive control, i.e. sciatic nerve (see Results section). Inserts: Human dermal fibroblasts react with the monoclonal antibodies TUJ (A) and 2G10 (B) as well as the polyclonal TUBB3 antibody (C). Cell nuclei are stained blue with DAPI. Scale bar = 100 μm (A-C). (TIF) [file pone.0145235.s004.tif]

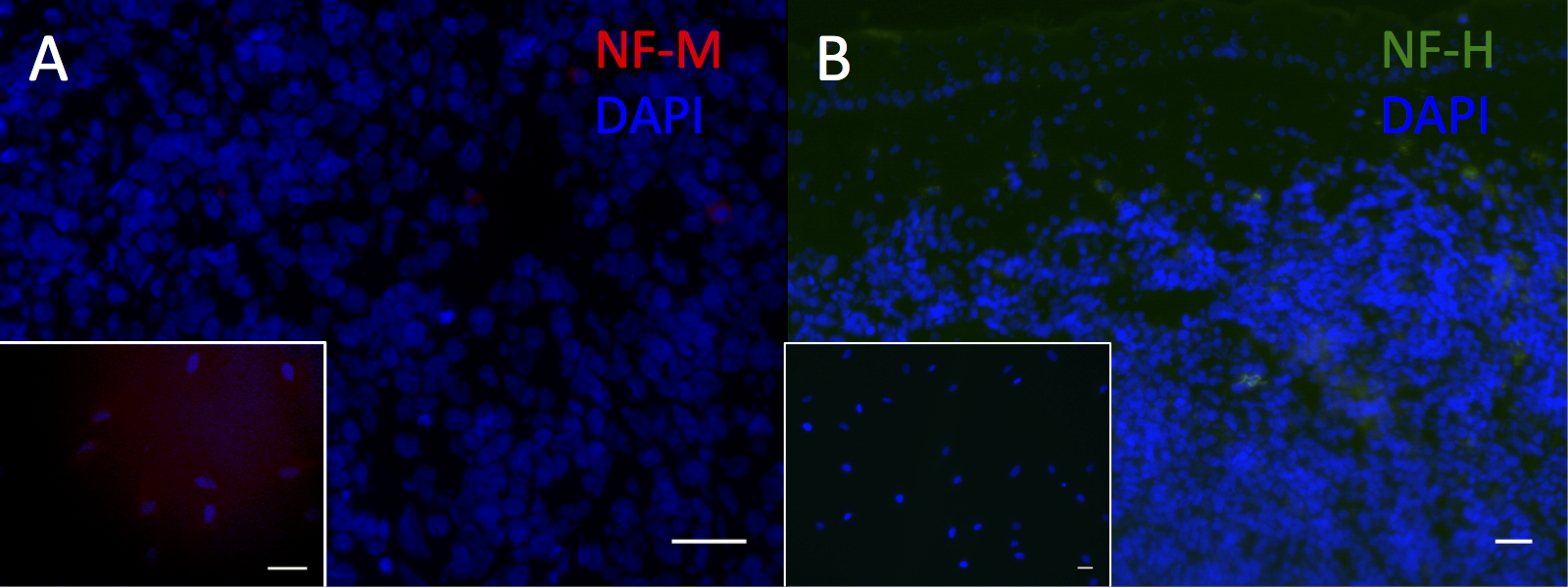

Supplement: S5 Fig — Cells in the dermis are negative for NEFM (A), although NEFH (B) immunostaining results in some faintly positive cells located beneath the epidermis, which may represent intradermal nerve endings. Inserts: Human dermal fibroblasts are negative for both NEFM (A) and NEFH (B). Cell nuclei are stained blue with DAPI. Scale bar = 50 μm (A) and 100 μm (B). (TIF) [file pone.0145235.s005.tif]
